# The Treatment of Typhoid Fever

**Published:** 1892-10-22

**Authors:** 


### ST. BARTHOLOMEW'S HOSPITAL.

### The Treatment of Typhoid Fever.

The treatment of typhoid fever will be dealt with under four heads, namely: I. Nursing; II. Diet; III. Drugs; and IV. Direct Antipyretic Treatment.

I. *Nursing.*—At St. Bartholomew's patients with most diseases are nursed on a flock mattress, which is made morning and evening; but enteric fever patients are always placed, from the first, on a spring mattress, and mainly for two reasons, one being that less disturbance of the patient is necessary, the other that by this means the great danger of the formation of bedsores is more easily avoided.

To this end also several details are carefully attended to. First, the patient is kept scrupulously clean and dry, and those parts most exposed to pressure, such as the sacrum and the hips, are kept from becoming sore by application either of brandy, or a lotion consisting of rectified spirit, and solution of alum about gr. xx. to the ounce.

In order to obtain a complete dryness, some dusting powder, consisting of starch, with perhaps the addition of a little zinc oxide or alum powder, is used freely. Then the patient is never allowed to lie too long in one position, as this would cause injurious pressure on one part, so he is turned into a different position every two or three hours, or oftener if necessary. This is especially important in the third week, when the patient lies like a log, and often resents any interference. If, in spite of this careful treatment, the skin should show signs of giving way, a water-bed is at once obtained, and the reddened part still further protected by means of air or water cushions, so managed as to take off the pressure. If a sore occurs it is treated with lead lotion, and a pad of amadou (with an opening for the sore), so as to keep off pressure. Later on, when the slough has come away, some stimulating application, such as Unguentum Resinae is used. Poultices are, if possible, avoided. The treatment of the mouth must be mentioned here, under the head of nursing. When it becomes dry, and the teeth and lips get covered with sordes, these are removed by washing out the mouth two or three times a day, aided by a soft brush or piece of soft rag, and then the lips and tongue are painted over with Mel. Boracis, or something else of the same kind, to prevent them from again rapidly drying and becoming sore.

II. *Diet.*—It need hardly be said that all typhoid patients are at once put on a fluid diet, and what one is accustomed to notice as the heading as to diet on their boards is: "D. L." (Dieta lactis, milk diet), followed by "no solid food," and perhaps an extra pint of milk. This means practically one pint of beef tea and two pints of milk. They are fed at intervals of about two hours and get about half a pint of fluid each time, which consists of milk diluted with about a third its bulk of water. Very commonly the beef tea above mentioned is replaced by milk, so that nothing else is given. In very severe cases food is given more frequently than every two hours, and in much smaller quantities than half a pint at a time. In cases, too, in which milk, even when diluted, does not agree, whey is substituted. This can be prepared by heating half a pint of fresh milk to about 140°F., and adding ʒi of Fairchild's essence of pepsin and stirring just enough to mix. The mixture is then allowed to stand until it coagulates; the curd is beaten up, and then it is strained. Perhaps the more common method of preparation, however, is to use one of the solutions of rennet, which can be easily obtained; directions for use are given on the bottle. Whey is much more easily digested in almost all cases than even well-diluted milk. Very often at the latter end of the third week, when the patient becomes very feeble, and milk, perhaps, becomes obnoxious to him, some mutton or beef essence, as made at St. Bartholomew's (that is to say, by stewing down lean parts of the meat at a low temperature, and then straining) is given as well. A few words must now be said as to returning to solid food after the fever has subsided. This is always put off as long as possible; but patients, who have been quite happy on fluid diet while the temperature is up, complain bitterly of being starved so soon as the temperature has fallen. The general rule, however, is that no solid food should be given until the temperature has remained normal or sub-normal for a week. In the meantime the patient is sometimes satisfied by giving leave for him to have a custard pudding, which, as one of the physicians remarks, is really fluid food.

At the end of a week solid diet is generally begun by giving bread and milk, made without the crust, and the crumb being finely grated. After this, the patient goes gradually on to ordinary diet, the effect of each advance in that direction being carefully watched.

III. *Drugs.*—Many typhoid patients require practically no treatment by drugs. At St. Bartholomew's they are generally given a weak solution of Hydrochloric or Nitrohydrochloric acid [illegible] to an ounce of water. This is perhaps more of a placebo than anything else. Many, however, almost always give some antipyretic drug if the temperature rises above 103° F., which, of course, it almost always does. Antipyrin is the drug most commonly used, and its object is, if possible, to increase the daily remissions of temperature and to make them last as long as possible; for this reason it is often given in a dose, say, of twenty grains, just when the thermometer shows a commencing fall, as all antipyretic drugs seem at this time to act with more effect, or it is given regularly in ten grain doses every six hours. Quinine and antipyrin are also given in cases in which the temperature rises above 104°; but whenever they are given the condition of the patient must be carefully watched, as the antipyrin especially tends to depress the heart.

For diarrhœa, recourse is almost always had to enemata of starch and opium, half a drachm or more of laudanum being used to each injection. In obstinate cases the pill of lead and opium of the Pharmacopœia is used as well.

Constipation is not interfered with unless giving a good deal of discomfort, or perhaps causing the temperature to keep up; it is then relieved by means of a soap and water enema. If there be much tympanites, a turpentine stupe, made by sprinkling freely with the oil a flannel wrung out of very hot water, and then applying it to the abdomen, often gives much relief.

As to stimulants, at St. Bartholomew's brandy is almost always what is used, and it is given in quantities of from one or two to six or eight ounces, the special indication for it being the condition of the heart, as shown by the softness and rapidity of the pulse and the weakness of the cardiac first sound. Other indications are a low, muttering kind of delirium and dry tongue; the quantity to be given is estimated by the effect produced.

As to the treatment of complications, a little must now be said. Bronchitis can hardly be called a complication, as it is practically always present, and frequently requires no direct attention. If it should give much trouble, a mixture containing some Carbonate of Ammonia and Senega is most commonly prescribed.

The two most important, because most frequent and dangerous, complications are perforation and hæmorrhage. For the former but little can be done. The sufferer is kept at absolute rest, and as quiet as possible, and to attain this end the more surely opium, either in the form of the tincture or the pill, is freely given, at frequent intervals. Operative treatment is now proposed for this almost inevitably fatal complication, but has never yet been attempted, we believe, at St. Bartholomew's. For hæmorrhage also the main treatment is opiates, to obtain, as far as possible, cessation of peristalsis, and, in addition, lead is given in the form of a pill with opium, and sometimes turpentine is given in doses of from ten to thirty minims in mucilage. Besides these, an ice-bag may be applied to the abdomen.

As soon as the temperature is down the stimulants are reduced in quantity, wine replacing brandy, and then some ordinary form of tonic is given, such as one containing iron, nux vomica, and quinine.

IV.—*Direct Antipyretic Treatment.*—This is the treatment *par excellence* for high temperature, and is applied in various ways, according to the amount of effect desired. The most simple method used is to give the patient what is practically an air bath, by removing all blankets and leaving him simply covered by bed-cradles, over which are spread sheets; this often assists very much in bringing down the thermometer Another way is by cold sponging; this may be combined with the above method, and is always used if the temperature rises much above 103 deg. If the thermometer still goes up to above 104·5 deg. the bath is resorted to. The patient is lifted into it on a sheet and covered by a blanket. The temperature of the water at first is usually about 90 deg., and by means of ice and cold water it is reduced to about 65 deg., the effect on the patient being watched by a thermometer in the rectum; some brandy must be at hand in case the heart should fail. When the temperature has fallen two or three degrees the patient is taken out, as it will be sure to fall more subsequently. This method of treatment may be almost said to be the usual treatment abroad for all typhoid cases. The same principle may be applied, with perhaps less difficulty, by wet packing with a sheet wrung out of ice water, or by placing ice in bags alongside the patient. An icebag to the head is frequently used, also in cases in which much delirium or headache is present.

A word or two in closing must be said as to the dejections of the patient. As they are the prime sources of contagion, they are always treated with a strong disinfectant, such as 1 in 20 solution of carbolic acid before being thrown into the drains, and all soiled linen is also looked upon as dangerous, until properly purified.

